# Preparation and In Vitro/In Vivo Characterization of Polymeric Nanoparticles Containing Methotrexate to Improve Lymphatic Delivery

**DOI:** 10.3390/ijms20133312

**Published:** 2019-07-05

**Authors:** Ji-Hun Jang, Seung-Hyun Jeong, Yong-Bok Lee

**Affiliations:** College of Pharmacy, Chonnam National University, 77 Yongbong-ro, Buk-Gu, Gwangju 61186, Korea

**Keywords:** methotrexate, PLGA, nanoparticles, lymphatic delivery

## Abstract

Methotrexate (MTX) is a folic acid antagonist used as an effective drug to treat various kinds of cancers. However, MTX has limited use in cancer chemotherapy due to its adverse effects such as poor bioavailability, low specificity, drug resistance, and dose-dependent side effects. To improve lymphatic delivery and reduce toxicity of MTX, MTX-loaded nanoparticles (NPs) were prepared in the present study. NPs were prepared with double emulsion solvent evaporation method using poly(lactide-co-glycolide) (PLGA). NPs were assessed for size, encapsulation efficiency, morphology, Fourier-transform infrared spectroscopy, X-ray diffraction, and thermal characterization. In vitro release profiles and cytotoxicity of these NPs were also evaluated. Prepared NPs and free MTX were administered orally or intravenously (5 mg/kg as MTX) to rats to evaluate their pharmacokinetic characteristics and lymphatic delivery effects. Mean particle size and encapsulation efficiency of NPs were 163.7 ± 10.25 nm and 93.3 ± 0.5%, respectively. Prepared NPs showed a sustained release profile of MTX in vitro and may be effective to cancer cells. Area under the blood concentration-time curve, total clearance, half-life, and lymphatic targeting efficiency were significantly different (*p* < 0.05) between prepared NPs and free MTX. These results demonstrate that MTX-loaded PLGA NPs are good candidates for targeted delivery of MTX to the lymphatic system.

## 1. Introduction

Nanomedicines have recently become an object of great interest in the area of drug delivery systems. Nanoparticles (NPs) are tiny materials of nanometer scale and have unique properties in the body in relation to their size. In addition, NPs can have a relatively large loading capacity due to their high surface area-to-volume ratio [1]. NPs lead to improve therapeutic efficiency and decrease side effects [2]. In addition, these systems can reduce drug doses by preventing rapid degradation or metabolism of drug and increasing drug concentration in the target tissue [3,4]. Using the properties of NPs, various attempts to nanoparticulate drugs have continued from the past to the present. Especially, drugs with many physicochemical barriers have been studied to overcome their limitations using nanocarrier drug delivery system.

**Figure 1:**
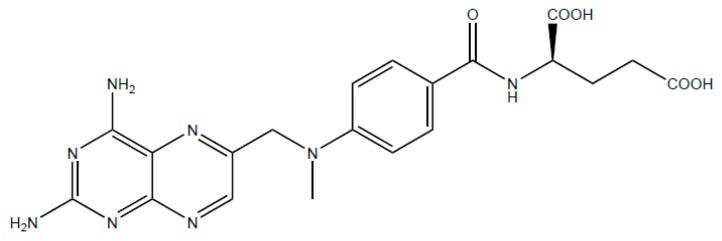
Chemical structure of methotrexate.

Methotrexate (2,4-diamino-N10-methyl propylglutamic acid, MTX) (Figure 1) is a folic acid antagonist that is widely used as an effective therapeutic agent to treat many solid tumors such as osteosarcoma, leukemia, lung cancer, and breast cancer [5,6]. In addition, it is used to treat autoimmune and inflammatory diseases such as rheumatoid arthritis, Crohn’s disease, multiple sclerosis, and psoriasis [7,8]. MTX is a weak dicarboxylic acid with a molecular weight of 454.5 g/mol. It possesses pKa values of 4.7–5.5 and low permeability (ClogP = 0.53) with poor aqueous solubility (0.01 mg/mL) [9]. MTX acts as an anticancer agent by inhibiting dihydrofolate reductase (DHFR), resulting in preventing the conversion of dihydrofolate (DHF) to tetrahydrofolate (THF) which is required for the synthesis of DNA and RNA. Influx of MTX into the cytosol ultimately leads to cell death [10,11,12].

Although MTX is used frequently as an effective drug for treating various tumors, autoimmune diseases, and inflammatory diseases, it has some limitations that restrict its use. First, bioavailability of MTX is low due to its poor water solubility and low permeability [9,13] as mentioned above. In addition, large amounts of MTX are eliminated by kidney within a short time, resulting in its short half-life and low drug concentration in target tissues [6,14]. Moreover, multi-drug resistance in cancer cells, dose-related toxicity and tissue toxicity including nephrotoxicity, hepatotoxicity, ulcerative colitis are known to be adverse effects of MTX [15,16].

In order to overcome these limitations of MTX, various attempts using drug delivery systems have been carried out. Pignatello et al. [17] have prepared liposome with MTX/lipoamino acids conjugate to overcome cellular resistance. However, cell inhibitory activity of liposome is lower than that of free MTX. Jeong et al. [18] have synthesized polymeric micelles using methoxy poly(ethylene glycol)-grafted chitosan copolymer. They found that the release of MTX from polymeric micelles was slower than that of free MTX. However, cytotoxicities of MTX and MTX-incorporated polymeric micelle to CT26 tumor cells were not significantly different [18]. In other studies, various drug carrier systems of MTX, including microparticle [19], microspheres [20,21], micro-emulsion [22,23], solid lipid nanoparticles [24], dendrimer conjugate [25], and polymersome [26] have been reported. However, most studies evaluated in vitro cytotoxicity. 

The lymphatic system plays an important role in cancer metastasis and immune response [27,28]. As mentioned above, MTX is an anti-cancer and immune-related agent. The delivery of MTX to lymphatic system is closely related to the immune system. Nano-sized drugs can be transferred to the lymphatic system through diffusion into the intercellular space of porous lymphatic walls [29,30,31]. Improved target delivery of MTX with nanoscale drug carriers can reduce the dose of MTX needed for the patient and maximize its therapeutic effect, thus reducing side effects. 

Researches have studied polymer-based NP in a variety of ways. Various studies have demonstrated that polymeric NPs of drugs have better cytotoxic effects than free drugs [32,33,34]. However, there is a limit to obtain consistent results in vivo. To the best of our knowledge, studies on pharmacokinetics (PKs) and delivery of prepared formulations to lymphatic system or target/normal tissues are limited. Thus, the objective of this study was to prepare MTX-loaded NPs to improve bioavailability and selectivity of MTX and reduce its toxicity by both oral and intravenous (IV) administration routes. We also compared PKs and lymphatic targeting efficiency of MTX-loaded NPs and free MTX in rats.

## 2. Results and Discussion

### 2.1. Preparation of MTX-Loaded NPs

#### 2.1.1. Determination of Optimal Concentration of PLGA in Internal Oil Phase

Poly(d,l-lactide-co-glycolide) (PLGA) is one of the most used biodegradable polymers. It is hydrolyzed to lactic acid and glycolic acid. These two monomers can be metabolized easily in the body by Krebs cycle. Owing to its minimal systemic toxicity, PLGA is widely used for drug delivery or biomaterial applications [35,36]. To evaluate the effect of PLGA polymer concentration on particle size, various concentrations of PLGA ranging from 0.5 to 8% (*w/v*) were used. As seen in Table 1, particle size increased as PLGA concentration increased gradually at a typical concentration of poly vinyl alcohol (PVA) and oil:water phase ratio. There were significant differences (*p* < 0.05) in particle size between different concentrations of PLGA except between 0.5 and 1% (*w/v*) of PLGA. The smallest particle size was 110.5 ± 8.38 when PLGA concentration was 0.5% (*w/v*). Based on these results, we selected 0.5% (*w/v*) as the optimal PLGA concentration which showed favorable particle size.

#### 2.1.2. Determination of Optimal Concentration of PVA in External Aqueous Phase

PVA is one of the most commonly used emulsifiers to stabilize particles [37]. A number of hydroxyl groups in PVA can hydrate with the surface of NPs to stabilize NPs. In addition, hydrocarbon chains in PVA could be adsorbed onto the surface of NPs [38]. Different concentrations of PVA ranging from 1 to 15% (*w/v*) were used to evaluate the effect of surfactant concentration on particle size. It was observed that mean particle size increased at increasing PVA concentration from 1 to 15% (*w/v*) at a typical PLGA concentration and oil:water phase ratio (Table 2). Such increase in particle size was significant (*p* < 0.05). When PVA concentration was 1% (*w/v*), the particle size was 135.5 ± 7.92 nm which was the smallest. These results suggested that the optimal concentration of PVA was 1% (*w/v*) which showed favorable particle size.

#### 2.1.3. Determination of Optimal Oil–Water Phase Volume Ratio

The effect of oil to water volume ratio on particle size was evaluated by varying this ratio from 1:1 to 1:6 (*v/v*). In prior test, we selected the optimal concentration of PLGA and PVA (0.5% and 1% *w/v*, respectively) having a particle size under 200 nm. As shown in Table 3, particles with the smallest size (103.0 ± 11.74 nm) were obtained when the volume ratio of oil and water was 1:3 (*v/v*). There were significant differences in particle size between various oil–water volume ratios (*p* < 0.05).

### 2.2. Characterization and Evaluation of NPs

#### 2.2.1. Particle Size, Zeta Potential, Drug Encapsulation Efficiency and Drug Loading

Particle size, zeta potential, drug encapsulation efficiency (EE), and drug loading (DL) of MTX-loaded PLGA NPs were evaluated by varying the amount of MTX in NPs (Table 4). NP size between 20 and 200 nm is considered appropriate. Small NPs with diameter less than 20 nm can be cleared through renal filtration [39]. Large particles with diameter more than 200 nm can be rapidly eliminated by the reticuloendothelial (RES) system [40]. Mean size of MTX-loaded NPs increased with increasing amount of MTX. In case of 3 mg of MTX, its particle size was the smallest (163.7 ± 10.25 nm). Difference in particle size was significant (*p* < 0.05) between MTX-loaded NPs with different amounts of MTX. It is known that zeta potential influences charge stability and electrostatic interactions within a suspension [41,42]. Zeta potentials of MTX-loaded PLGA NPs ranged from −17.3 ± 2.63 to −20.4 ± 1.54 mV, showing no significant difference (*p* > 0.05) in zeta potential among MTX-loaded NPs with different amounts of MTX. EE (%) and DL (%) of MTX-loaded NPs were generally lower with increasing amount of MTX. Differences in EE and DL among MTX-loaded PLGA NPs with different amounts of MTX were statistically significant (*p* < 0.05). When the amount of MTX was 3 mg, EE (%) and DL (%) were 93.34 ± 0.51 and 15.45 ± 0.34%, respectively, which were the highest. Based on these results, 3 mg of MTX was finally selected as the optimal amount for preparing of NPs.

#### 2.2.2. Fourier-Transform Infrared (FTIR) Spectroscopy, X-ray Diffraction (XRD) Analysis and Thermal Analysis

**Figure 2:**
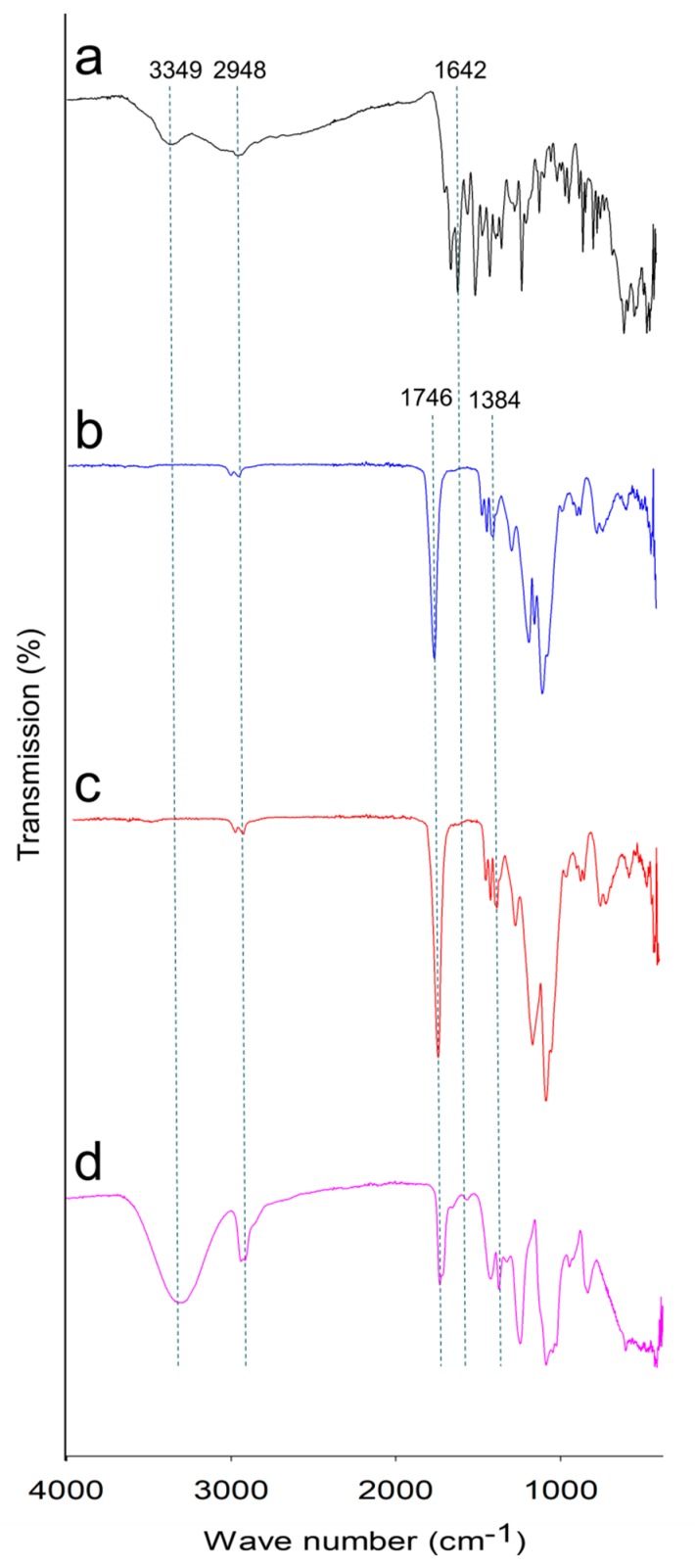
Fourier-transform infrared (FTIR) spectra of methotrexate (MTX) (**a**), poly(d,l-lactide-co-glycolide) (PLGA) (**b**), blank nanoparticles (NPs) (**c**), and MTX-loaded PLGA NPs (**d**).

Fourier-transform infrared (FTIR) spectroscopy was performed to demonstrate the chemical structure of compounds. FTIR spectra of MTX, PLGA, and MTX-loaded PLGA NPs (0.5% (*w/v*) of PLGA, 1% (*w/v*) of PVA, 1:3 (*v/v*) of oil–water phase volume ratio) are presented in Figure 2. Pure MTX had absorption peaks at 3349, 2948, and 1642 cm^−1^ indicating the presence of –OH, C–H, and C=C functional groups, respectively. Absorption peaks of PLGA were observed at 2948, 1746, and 1384 cm^−1^ reflecting C–H, C=O and C–O groups, respectively. Typical peaks of PLGA were observed in the spectra of blank NPs, with no considerable additional peaks. In addition, characteristic spectra of MTX-loaded PLGA NPs have the absorption peaks similar to MTX and PLGA, showing no additional peaks compared to the spectra of MTX and PLGA. Therefore, we could conclude that there was no chemical interaction between MTX and polymers. 

**Figure 3:**
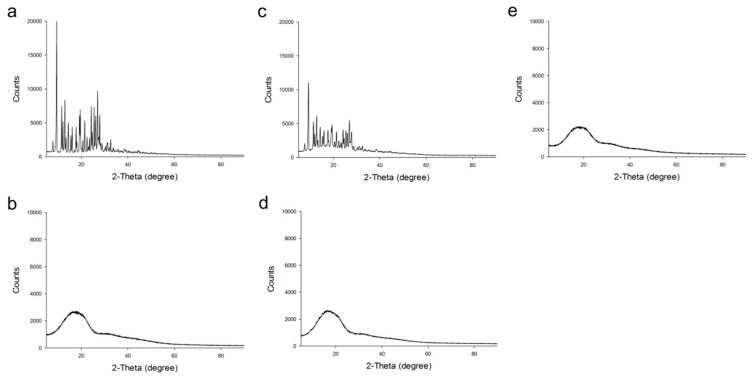
X-ray diffraction (XRD) patterns of MTX (**a**), PLGA (**b**), physical mixture of MTX/PLGA (**c**), blank NPs (**d**), and MTX-loaded PLGA NPs (**e**).

X-ray diffraction (XRD) was analyzed to investigate drug crystallinity. Figure 3 shows XRD spectra of MTX, PLGA, physical mixture of MTX/PLGA (1/4, *w/w*), and MTX-loaded PLGA NPs (0.5% (*w/v*) of PLGA, 1% (*w/v*) of PVA, 1:3 (*v/v*) of oil–water phase volume ratio). Pure MTX exhibited multiple intense peaks. PLGA showed different patterns without distinct peaks. For physical mixture of MTX/PLGA, its spectra appeared to be a mixture of pure MTX and PLGA spectra. However, peaks of MTX-loaded NPs were similar to the aspect of PLGA, showing no characteristic peaks of MTX. In addition, XRD patterns of blank NPs were also similar to PLGA and MTX-loaded NPs, with no additional characteristic peaks. These results suggested that MTX was entrapped in PLGA NPs as an amorphous or disordered-crystalline state. As MTX is encapsulated into PLGA NP, it is present in an amorphous or molecularly dispersed form [43,44]. Therefore, the characteristic patterns of crystalline form do not appear.

**Figure 4:**
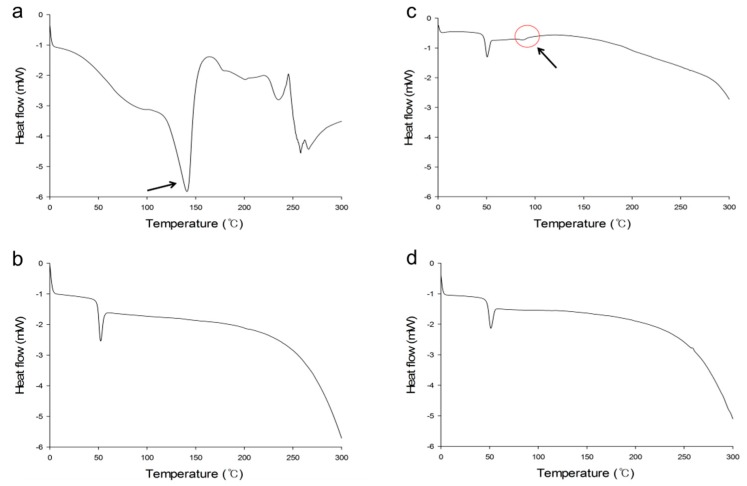
Differential scanning calorimetry (DSC) thermograms of MTX (**a**), PLGA (**b**), physical mixture of MTX/PLGA (**c**) and MTX-loaded PLGA NPs (**d**). ↖: endothermic melting peak of MTX.

Differential scanning calorimetry (DSC) studies were performed to characterize physical properties of compounds including melting points. Thermograms of MTX, PLGA, physical mixture of MTX/PLGA (1/10, *w/w*), and MTX-loaded PLGA NPs (0.5% (*w/v*) of PLGA, 1% (*w/v*) of PVA, 1:3 (*v/v*) of oil–water phase volume ratio) are shown in Figure 4. In the thermogram of pure MTX, there was an endothermic melting peak at 140 °C. The endothermic peak of PLGA appeared at 50 °C, indicating glass transition temperature. Physical mixture of MTX and PLGA exhibited peaks similar to characteristics of MTX and PLGA. On the other hand, MTX-loaded NPs showed an endothermic peak at 50 °C, a characteristic peak of PLGA. However, melting peak of MTX was not observed. These results indicated that MTX was well encapsulated in PLGA NPs as an amorphous or disordered-crystalline state. As mentioned above, MTX loaded in PLGA NP loses crystallinity. Therefore, the characteristic endothermic melting peak of crystalline form is not observed [44,45]. These results are consistent with the results of XRD analysis. 

#### 2.2.3. Morphological Studies

**Figure 5:**
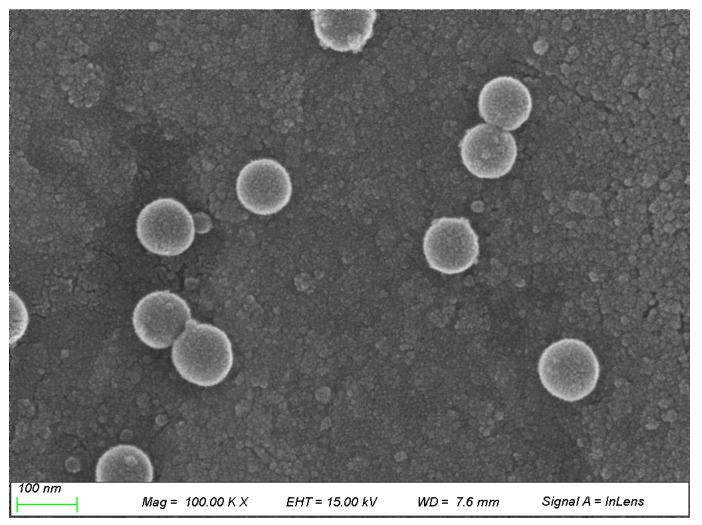
Scanning electron microscope (SEM) image of MTX-loaded PLGA NPs.

Morphology of MTX-loaded PLGA NPs (0.5% (*w/v*) of PLGA, 1% (*w/v*) of PVA, 1:3 (*v/v*) of oil–water phase volume ratio) was characterized using scanning electron microscope (SEM). As shown in Figure 5, prepared NPs were observed in spherical form with a relatively narrow distribution. Polydispersity index (PDI) indicating the degree of non-uniformity of a size distribution of particles was calculated to be 0.21. PDI values ranged from 0.0 (for a highly monodisperse sample) to 1.0 (for a highly polydisperse sample with multiple particle size populations). PDI values of 0.2 or less are generally acceptable in practice for polymer-based NP materials [46].

#### 2.2.4. In Vitro Cytotoxic Studies

**Figure 6:**
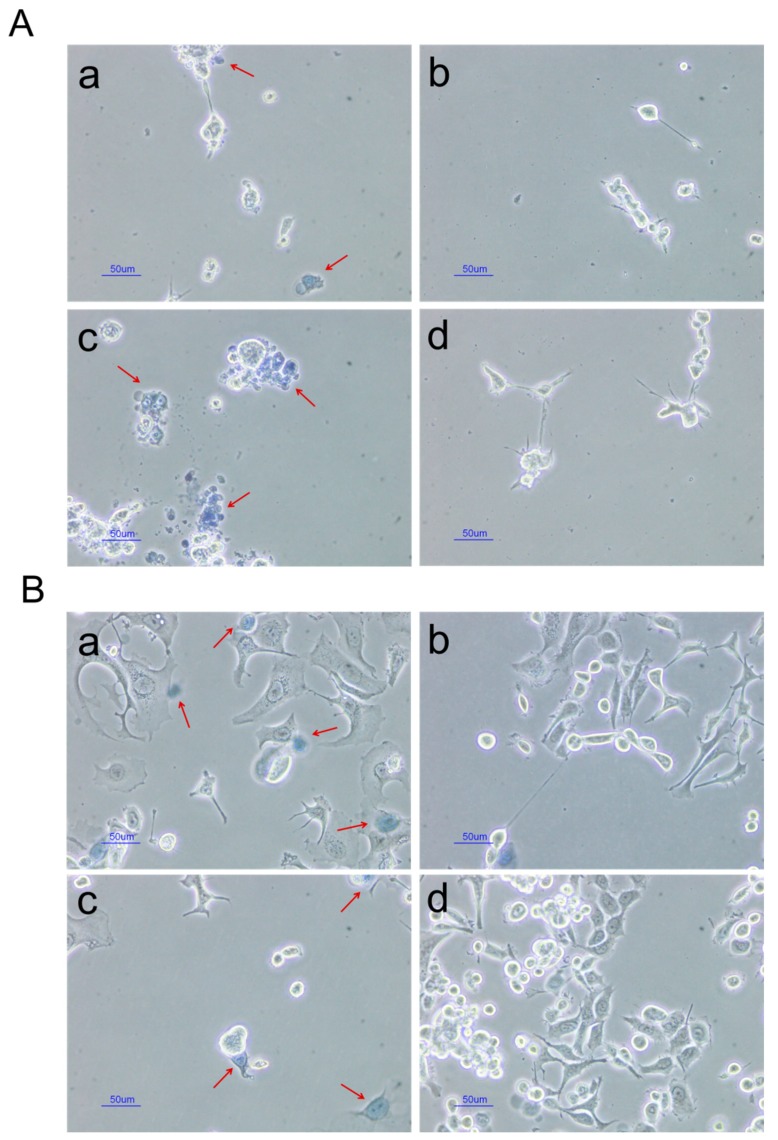
Results of trypan blue assay after treating CWR22Rv1 cells (**A**) and MCF-7 cells (**B**) with free MTX (**a**, 0.01 mg/mL), blank NPs (**b**), MTX-loaded PLGA NPs (**c**, 0.01 mg/mL as MTX), or control (**d**). ↖: trypan blue stained cells.

Trypan blue method is one of the commonly used assays for evaluating cell viability. The principle of this method is that dead cells can absorb trypan blue into the cytoplasm because of loss of membrane selectivity, whereas live cells remain unstained [47,48,49]. Morphologies of CWR22Rv1 cells and MCF-7 cells after treatment with free MTX (0.01 mg/mL), blank NPs, and MTX-loaded NPs (0.01 mg/mL as MTX) were compared. Results are shown in Figure 6. Stained cells (red arrow) representing dead cells were observed more frequently in free MTX and MTX-loaded NPs groups. Cell morphology also changed to a round shape in these groups. Therefore, free MTX and MTX-loaded NPs were considered to be cytotoxic. In addition, stained cells were hardly visible in the blank NP group, indicating that PLGA was not cytotoxic. 

**Figure 7:**
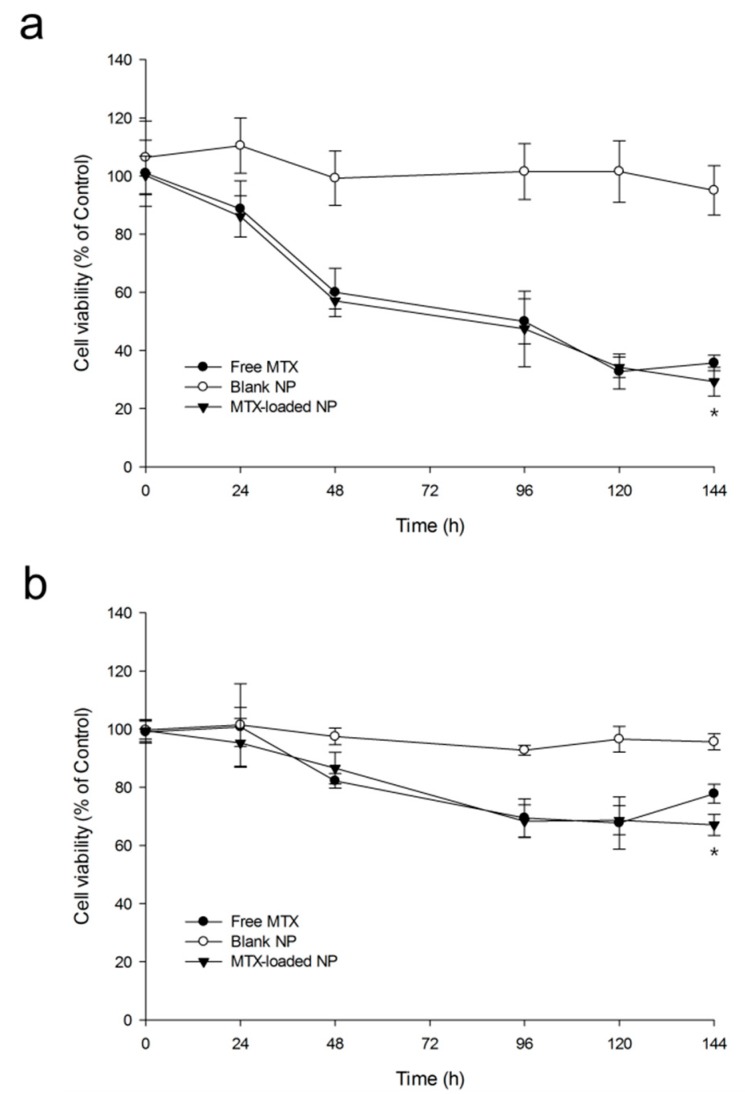
Viabilities of CWR22Rv1 cells (**a**) and MCF-7 cells (**b**) after treated with free MTX (0.01 mg/mL), blank NPs, or MTX-loaded NPs (0.01 mg/mL as MTX) (*n* = 3, mean ± SD). * *p* < 0.05 between prepared PLGA NPs and free MTX groups.

Figure 7 shows cell viability-time profiles of CWR22Rv1 cells and MCF-7 cells treated with free MTX (0.01 mg/mL), blank NPs, or MTX-loaded NPs (0.01 mg/mL as MTX) (*n* = 3). In blank PLGA NPs, the viability of both cells did not decrease to be below 90% for 144 h. This demonstrated that components constituting NPs, including the polymer, did not affect cytotoxicity. Cell viabilities of free MTX and MTX-loaded NP groups showed about 70% reduction for CWR22Rv1 cells and 35% reduction for MCF-7 cells. Although viabilities of both cells in free MTX and MTX-loaded NP groups were decreased, such decreases were not significantly different for up to 120 h. The rate of cell viability steadily decreased in the MTX-loaded NP group while the rate in the free MTX group was slightly increased at 144 h. MTX is known to have dose- and time-dependent effects on inhibition of cell proliferation and induction of apoptosis [50]. However, MTX concentrations in free MTX and NPs were the same in this experiment, so only time-dependent effects remain. Thus, the drug effects up to 120 h are similar between free MTX and NPs. These results suggested that NP formulation allowed for more controlled and sustained release of MTX.

Considering the mechanism of MTX, intracellular folates and MTX were quantified as an alternative method to assess cytotoxicity. We quantified absolute concentrations of MTX, DHF, and THF in CWR22Rv1 cells and MCF-7 cells after incubating cells with free MTX, blank NPs, or MTX-loaded PLGA NPs for 144 h (Table 5). We also evaluated changes of pool sizes in DHF and THF. As a result of free MTX treatment, 4.25-fold increase of DHF with 2.16-fold decrease of THF in CWR22Rv1 cells and 1.88-fold increase of DHF with 1.76-fold decrease of THF in MCF-7 cells were found compared to control. In addition, treatment with MTX-loaded NPs resulted in 7.38- and 2.76-fold increase in DHF but 4.36- and 2.54-fold reduction in THF in CWR22Rv1 and MCF-7 cells, respectively. In contrast, blank NPs administration showed no significant difference in DHF and THF from control in both cell groups. Differences in DHF and THF between formulations suggest that intracellular DHFR activity is further inhibited when NPs are administered. Difference in intracellular MTX concentration between formulation indicated that PLGA NPs improved cellular uptake or sustained release of MTX.

#### 2.2.5. In Vitro Release Studies

**Figure 8:**
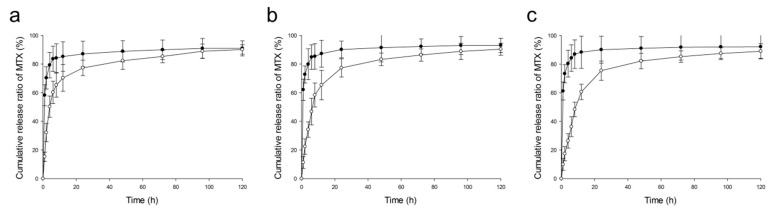
In vitro drug release profiles of free MTX (-●-) and MTX-loaded PLGA NPs (-○-) in various pH media. a, pH 1.2; b, pH 6.8; c, pH 7.4.

The in vitro drug release test was conducted to evaluate the stability of formulation in solution and the release profile of drug from prepared formulation at drug development stage. In vitro release profiles of MTX from PLGA NPs and free MTX as control at pH 1.2, 6.8, and 7.4 were compared. Results are shown in Figure 8. Both formulations exhibited total drug release of about 90% up to 120 h in all media. In case of free MTX, released amounts increased rapidly up to the first 6 h, reaching 90% within 24–48 h in all media. However, MTX-loaded PLGA NPs showed sustained release profiles compared to control in all media. An initial high release of MTX for the first 24 h was observed in all media. Released amounts exceeded 80% at 48 h and increased gradually thereafter. When release profiles were compared according to pH, the release rate of MTX at lower pH was faster than that at higher pH. Slopes of in vitro release profiles of MTX from PLGA NPs at pH 1.2, 6.8 and 7.4 up to the initial 6 h were 10.05, 7.81, and 6.05, respectively. This result could be explained with the results of previous studies [51,52]. Zolnik and Burgess [52] have reported that acidic pH conditions can accelerate drug release by affecting degradation of polymer. As a result of visual inspection, the color in pH 1.2 medium was the yellowest (the closest to MTX color) for the initial 6 h. Therefore, it could be concluded that these prepared MTX-loaded NPs have potential characteristics for controlled release in blood or gastrointestinal fluid.

#### 2.2.6. Determination of MTX in Rat Plasma, Lymph Node and Tissues

Concentrations of MTX in rat plasma, lymph node, and various tissues were determined by the UPLC-MS/MS method with modification of the previously reported MTX assay [53,54]. Some conditions such as mobile phase composition and sample preparation procedure were optimized for our system. The lower limit of quantification (LLOQ) was 0.1 ng/mL in the present study. It was lower than that of previous studies. The analytical method was validated for selectivity, sensitivity, linearity, precision, accuracy, recovery, matrix effect and stability according to FDA Bioanalytical Method Validation Guidance [55].

**Figure 9:**
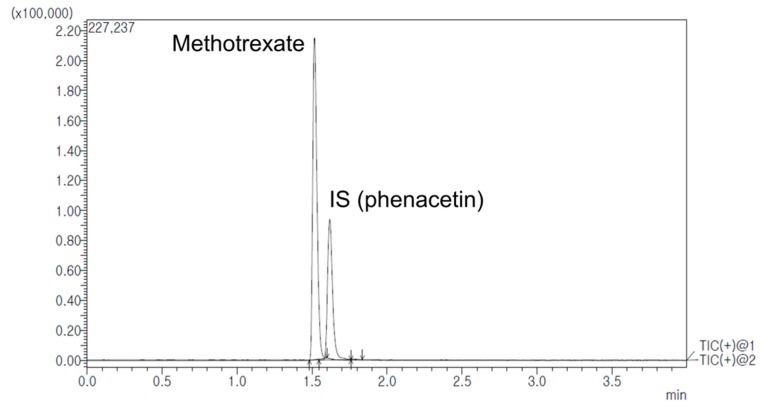
Representative chromatograms of MTX and internal standard (IS) obtained in multiple reaction monitoring (MRM) positive ion mode of UPLC-MS/MS.

**Figure 10:**
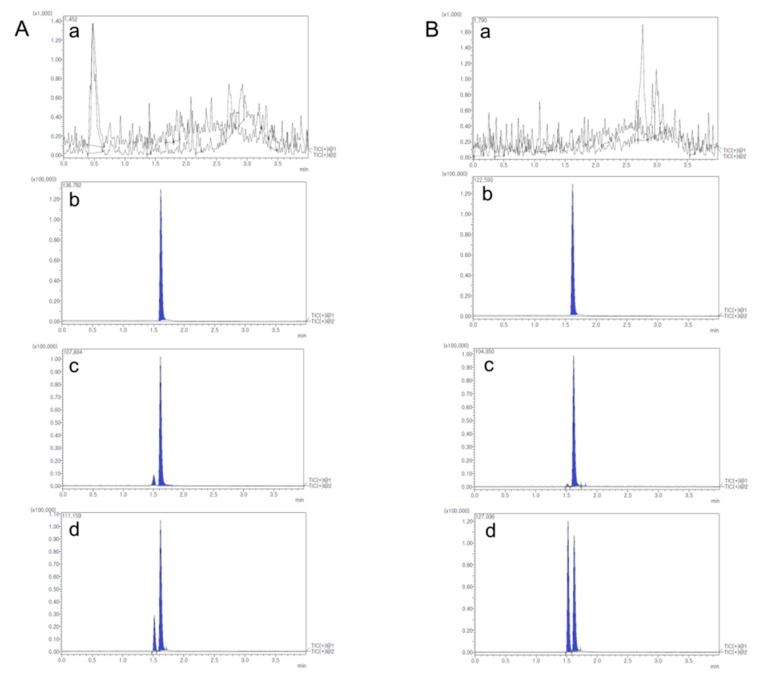
Chromatograms of blank rat biological samples (**a**), zero rat samples containing IS (**b**), blank rat samples containing lower limit of quantification (LLOQ) of MTX and IS (**c**), rat samples after administration of MTX (**d**). A, Rat plasma; B, Rat lymph node.

Quantitative analysis for MTX in rat biological samples was performed using a Shimadzu Nexera X2 Series UPLC system (Shimadzu Corp., Kyoto, Japan) coupled with a Shimadzu 8040 mass spectrometer (Shimadzu Corp., Kyoto, Japan). Optimized chromatographic separation of MTX was conducted with a KINETEX core-shell C18 column (50 mm × 2.1 mm, 1.7 μm particle size, Phenomenex, CA, USA) at a column temperature of 40 °C. The mobile phase was prepared with 0.1% (*v/v*) formic acid in water (mobile phase A) and acetonitrile (mobile phase B) with a gradient elution. The flow rate was 0.3 mL/min. Elution program was: 0–0.5 min (10% B), 0.5–0.8 min (10–90% B), 0.8–3.0 min (90% B), 3.0–3.01 min (90–10% B), and 3.01–4.0 min (10% B). Product ion mass spectrum of MTX was obtained in full scan mode after injecting individual standard solution into the mass spectrometer. MTX and internal standard (IS, phenacetin) generated a protonated molecular ion [M+H]^+^ in positive electrospray ionization mode. The most abundant fragment ion on multiple reaction monitoring (MRM) was m/z 454.7→308.1 for MTX and m/z 180.0→110.1 for IS. Figure 9 shows representative MRM chromatograms with retention times of 1.52 min for MTX and 1.62 min for IS. Selectivity was presented in responses of blank rat biological samples (a), zero rat samples containing IS (b), blank rat samples containing LLOQ of MTX and IS (c), and rat samples after administration of MTX (d). Representative chromatograms are shown in Figure 10. There were no significant interferences from endogenous substances around retention times of analytes in blank rat biological samples.

Linearities for MTX in rat biological samples were excellent over the concentration range of 0.1–1000 ng/mL. Regression equations from five replicate calibration curves for rat biological samples were: y = (0.0215 ± 0.0016)x−(0.0071 ± 0.0101) for rat plasma, y = (0.0203 ± 0.0013)x−(0.0022 ± 0.0009) for axillary lymph node, y = (0.0217 ± 0.0021)x + (0.0053 ± 0.0001) for mesenteric lymph node, y = (0.0209 ± 0.0018)x + (0.0036 ± 0.0012) for spleen, y = (0.0213 ± 0.0019)x + (0.0008 ± 0.0069) for thymus, y = (0.0227 ± 0.0022)x + (0.0034 ± 0.0017) for kidney, and y = (0.0223 ± 0.0019)x−(0.0018 ± 0.0002) for liver. These calibration curves fitted well, with the correlation coefficient (r^2^) exceeding 0.99. In regression equations, y was the peak-area ratio of MTX to IS and x (ng/mL) was the concentration of MTX.

All values of accuracy ranged from 96.47 to 104.15%, with a precision of < 7.74% for MTX in rat biological matrices. Extraction recoveries ranges of MTX in rat biological samples were 95.68–99.29%. There was no significant matrix effect on the detection of MTX (98.1–101.25%). Stabilities of MTX in rat samples at various conditions were also evaluated, showing 97.52–101.01%. 

#### 2.2.7. Pharmacokinetic Analysis and Targeting Delivery Evaluation

**Figure 11:**
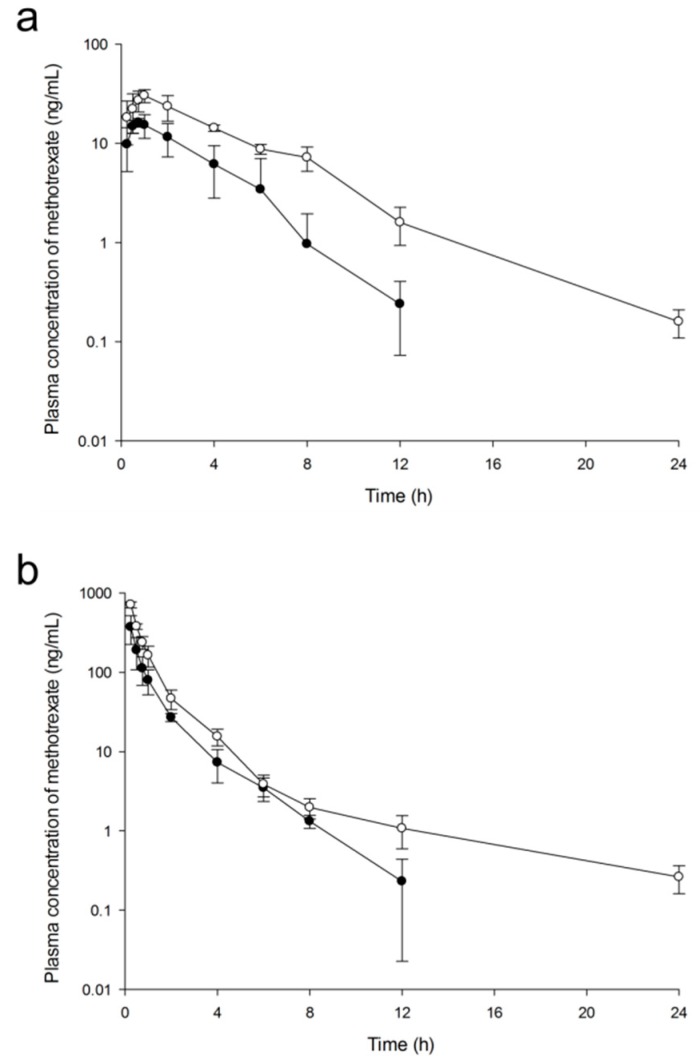
Mean plasma concentration-time profiles of MTX after oral (**a**) or intravenous (**b**) administration of free MTX (-●-, 5 mg/kg) and MTX-loaded PLGA NPs (-○-, 5 mg/kg as MTX) to rats. Vertical bars represent standard deviation of the mean (*n* = 5).

Figure 11 shows mean plasma concentration-time profiles of MTX after oral or IV administration of free MTX (5 mg/kg, *n* = 5) and MTX-loaded PLGA NPs to rats (5 mg/kg as MTX, *n* = 5). When MTX-loaded NPs were administered, MTX was absorbed more and remained longer in blood than that when free MTX was administered. PK parameters obtained by noncompartmental analysis are presented in Table 6. After oral or IV administration, the area under the plasma concentration-time curve (AUC), half-life (t_1/2_), maximum blood concentration (C_max_), systemic clearance (CL) and volume of distribution (V_d_) of MTX-loaded NPs were significantly different (*p* < 0.05) from those of free MTX. MTX-loaded NPs showed almost two times higher AUC, longer half-life, and two times lower CL than free MTX. These PK parameter differences were due to the fact that MTX-loaded NPs were particle-type molecules surrounded by PLGA, thus exhibiting different PK characteristics from free MTX which had drug-specific properties. The absolute oral bioavailability (F_ab_) value of PLGA NPs was 20.99%, different from that of free MTX at 16.04%. Relative bioavailability (F_rb_) value of PLGA NPs was 254.09% for oral administration and 194.19% for IV administration.

Our results of bioavailability value for free MTX were similar those of a previous study [56]. The reported bioavailability after oral or IV administration at a low dose of MTX (0.5 mg/kg) in rats was 12.5% in the fasted group and 30.0% in the non-fasted group. In addition, the previous study concluded that feeding (not-fasted) enhanced oral bioavailability of MTX in rats [56]. Our animal experiment conditions were similar to conditions of the fasted group. Kuroda et al. [57] have also reported PK parameters after oral or IV administration of 0.1, 0.5, and 2.5 mg/kg MTX in rats. In their study, oral bioavailability was obtained from 8.1 to 21%. It was estimated that about 76% of MTX administered orally to rats was eliminated due to incomplete absorption and metabolism in gastrointestinal tract and that 14% of MTX was eliminated by first-pass effect [57]. The reported time to reach C_max_ (T_max_) of MTX at oral dose was very short, ranging from 0.80 ± 0.12 h to 1.6 ± 0.7 h, similar to our experimental results (0.75 ± 0.25 h) of free MTX.

**Figure 12:**
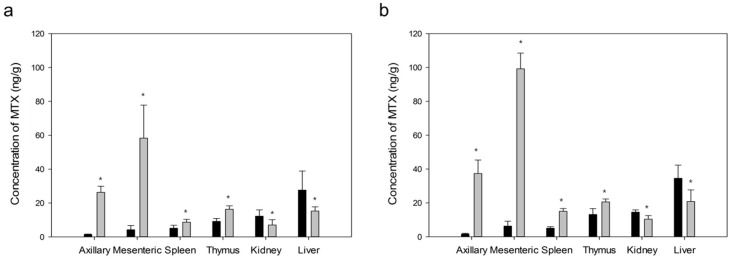
Concentrations of MTX in axillary and mesenteric lymph nodes, spleen, thymus, kidney and liver at 2.5 h after oral (**a**) or intravenous (**b**) administration of MTX-loaded PLGA NPs (■, 5 mg/kg as MTX) and free MTX (■, 5 mg/kg) to rats. Vertical bars represent mean ± SD (*n* = 5). *p < 0.05 between prepared PLGA NPs and free MTX groups.

Concentrations of MTX in axillary and mesenteric lymph nodes, spleen, thymus, kidney and liver after oral or IV administration are shown in Figure 12. MTX-loaded PLGA NPs showed higher concentrations of MTX in both lymph nodes than free MTX (*p* < 0.05). Concentrations of MTX from PLGA NPs were increased in spleen and thymus, but decreased in kidney and liver compared to those from free MTX (*p* < 0.05). These results indicated that improved lymphatic delivery of MTX from PLGA NPs was attributed to prolonged blood retention and decreased renal and hepatic uptake associated with metabolism and excretion in the body.

**Figure 13:**
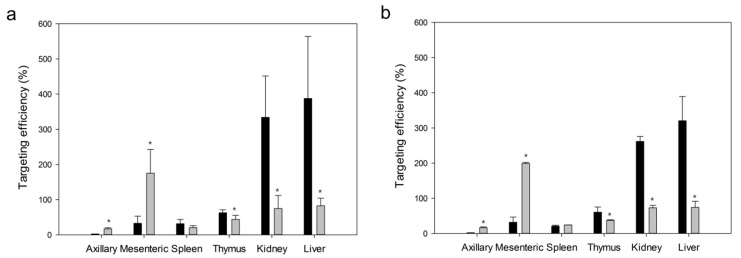
Targeting efficiencies of MTX to axillary and mesenteric lymph nodes, spleen, thymus, kidney and liver at 2.5 h after oral (**a**) or intravenous (**b**) administration of MTX-loaded PLGA NPs (■, 5 mg/kg as MTX) and free MTX (■, 5 mg/kg) to rats. Vertical bars represent mean ± SD (*n* = 5). * *p* < 0.05 between prepared PLGA NPs and free MTX groups.

Lymphatic targeting efficiencies of MTX into axillary and mesenteric lymph nodes, spleen, thymus, kidney and liver were obtained (Figure 13). Targeting efficiency was calculated as the ratio of the concentration in lymph nodes to the concentration in plasma. After oral or IV administration of MTX-loaded NPs, the targeting efficiency in lymph nodes was significantly different from that of free drug (*p* < 0.05). Lymphatic targeting efficiencies of prepared NPs were 5.3 to 8.5 times higher than those of free MTX. However, targeting efficiencies of MTX from PLGA NPs in thymus, kidney and liver were lower than those from free MTX (*p* < 0.05).

These results suggest that prepared MTX-loaded PLGA NPs are effective for lymphatic delivery of MTX. They are also useful for decreasing tissue toxicity such as nephrotoxicity and hepatotoxicity as major side effects of MTX for both administration routes.

As shown in Figure 11 and Table 6, MTX from PLGA NPs remains much longer in blood than free MTX with a longer half-life and lower CL. This can be attributed to the fact that PLGA polymer prevents MTX from hydrolysis or metabolism [58]. In addition, NPs with an appropriate size inhibit phagocytosis by the immune system, such as RES system and lead to less excretion into the kidney or liver [40]. These are consistent with the results of tissue distribution study.

The size of NP is the most important factor in the lymphatic delivery. In various studies, it has been reported that the delivery of particle-type agents to the lymphatic system is highly dependent on the particle size [59]. As mentioned in Section 1, nano-sized drugs are better able to migrate into lymphatic system or small capillary where larger particles cannot reach. However, particles smaller than 10 nm can be recirculated systemically [60]. Particles larger than 200 nm are slowly transited to the lymphatic system and this induced more removal by macrophages [61].

Finally, we conclude that polymeric NPs give physicochemical stability to protect MTX from excretion or degradation, thereby increasing blood retention time, which allows the appropriate sized NPs to migrate further to the lymphatic system.

Formulation studies of MTX using PLGA NPs have been actively conducted recently. However, many researches have focused on the physicochemical properties of formulation [62,63] or in vitro cellular uptake/cytotoxicity [34,64,65]. Several studies have also focused on the distribution to blood-brain barrier or central nervous system, which are difficult to access large particles [66,67,68]. However, we evaluated PK profiles in vivo as well as the physicochemical properties of the prepared formulations in this study. We also proposed a new approach to clinical application and therapeutic methods of NPs focusing on the improved delivery to the lymphatic system, which plays an important role in tumor metastasis and immune response. In addition, intracellular MTX and folates were quantitatively analyzed by in vitro cytotoxicity assay. The results revealed that drug loaded in NPs remained higher in tumor cells than free MTX, resulting in the higher cell death by folate pool changes. We first reported the toxic effects of MTX on cancer cells by quantifying intracellular MTX and folate pool through UPLC-MS/MS.

However, evaluation of therapeutic aspects such as lowering cancer cell transfer rate and increasing cancer survival rate lacked in this study. How increased lymphatic delivery of MTX might affect other side effects of MTX such as neurotoxicity and immunotoxicity was not assessed either. Therefore, further studies are needed to evaluate not only side effects, but also pharmacodynamic aspects using tumor-induced animal models.

## 3. Materials and Methods

### 3.1. Materials

MTX (purity ≥ 99%), PLGA with a molecular weight 30,000–60,000 g/mol, PVA with molecular weight of 31,000–50,000, phosphate buffered saline (PBS), DHF (purity > 90%) and THF (purity > 90%) were purchased from Sigma-Aldrich (St. Louis, MO, USA). UPLC-MS/MS grade methanol, acetonitrile, water (18.2 mΩ), and HPLC grade ethyl acetate were purchased from Fisher Scientific (Fair Lawn, NJ, USA). UPLC-MS/MS grade formic acid was supplied by Tokyo Chemical Industry (Tokyo, Japan). All other reagents and solvents were in analytical grades.

Roswell Park Memorial Institute (RPMI) 1640 medium and Trypsin-EDTA were obtained from Welgene Inc. (Gyeongsan-si, Republic of Korea). Fetal bovine serum (FBS) was supplied from Merck Millipore (Burlington, MA, USA). Penicillin/streptomycin was purchased from Thermo Fisher Scientific (Waltham, MA, USA). Trypan blue was purchased from Sigma-Aldrich (St. Louis, MO, USA). MCF-7 cells were purchased from Korean Cell Line Bank (Seoul, Republic of Korea). CWR22Rv1 cells were purchased from ATCC (Manassas, VA, USA).

### 3.2. Preparation of MTX-loaded NPs

MTX-loaded PLGA NPs were prepared by double emulsion solvent evaporation method of previously reports [65,69] with modification. Briefly, PLGA was dissolved in dichloromethane (3 mL) to prepare oil phase. MTX (3 mg) was dissolved in acetone (3 mL) and added to PLGA solution. The resulting solution was vortexed moderately and sonicated for 1 min. It was then added to PVA aqueous solution (9 mL) drop wise under stirring at a low speed. After that, 0.1% PVA aqueous solution (15 mL) was added to the resulting emulsion and stirred on a magnetic stirrer at 1200 rpm for 2 h. Obtained NPs were separated by centrifugation at 13,500× *g* for 15 min. The suspension was deep-frozen at −80°C for 12 h and lyophilized with vacuum pressure less than 50 mTorr at a temperature of −50°C for 48 h. The supernatant was also collected for analyzing free drug.

#### 3.2.1. PLGA Concentration in the Internal Oil Phase

The influence of PLGA concentration on particle size was determined by increasing its concentration from 0.5% to 8% (*w/v*). Tests were conducted under the condition that other variables were fixed (PVA 10% (*w/v*), oil−water phase volume ratio 1:3 (*v/v*)).

#### 3.2.2. PVA Concentration in the External Aqueous Phase

The influence of PVA concentration on particle size was determined by increasing its concentration from 1% to 15% (*w/v*). Tests were conducted under the condition that other variables were fixed (PLGA 4% (*w/v*), oil−water phase volume ratio 1:3 (*v/v*)).

#### 3.2.3. The Oil−water Phase Volume Ratio

The influence of volume ratio of oil−water phase on particle size was determined by using different ratios ranging from 1:1 to 1:6 (*v/v*). Concentrations of PLGA and PVA were selected from the prior test.

### 3.3. Characterization and Evaluation of NPs

#### 3.3.1. Particle Size and Zeta Potential

Mean particle size and size distribution analysis were performed using the dynamic light scattering technique (SZ-100, Horiba scientific, Kyoto, Japan) at 25°C. Zeta potentials (SZ-100, Horiba scientific, Kyoto, Japan) were measured to evaluate surface charge and stability of particles. These prepared NPs were diluted in deionized water (1/10, *w/v*) and placed in measurement cell for analysis (*n* = 5).

#### 3.3.2. Drug Encapsulation Efficiency and Drug Loading

For determination of EE and DL, NPs were separated by centrifugation at 13,500× *g* for 15 min. Free MTX was assayed from the collected supernatant. The amount of free MTX was determined by UPLC-MS/MS method. EE and DL were calculated with the following equations:

(1) $$\mathrm{EE}\;(\%)\;=\;\frac{\mathrm{amount}\;\mathrm{of}\;\mathrm{MTX}\;\mathrm{in}\;\mathrm{preparing}\;\mathrm{formulation}-\mathrm{amount}\;\mathrm{of}\;\mathrm{MTX}\;\mathrm{in}\;\mathrm{supernatant}}{\mathrm{amount}\;\mathrm{of}\;\mathrm{MTX}\;\mathrm{in}\;\mathrm{preparing}\;\mathrm{formulation}}\;\times 100$$

(2) $$\mathrm{DL}\;(\%)\;=\;\frac{\mathrm{amount}\;\mathrm{of}\;\mathrm{MXT}\;\mathrm{in}\;\mathrm{preparing}\;\mathrm{formulation}-\mathrm{amount}\;\mathrm{of}\;\mathrm{MTX}\;\mathrm{in}\;\mathrm{supernatant}}{\mathrm{polymer}\;\mathrm{weight}\;+\mathrm{amount}\;\mathrm{of}\;\mathrm{MTX}\;\mathrm{in}\;\mathrm{preparing}\;\mathrm{formulation}}\;\times 100$$

#### 3.3.3. Fourier-Transform Infrared (FTIR) Spectroscopy, X-ray Diffraction (XRD) Analysis and Thermal Analysis

MTX, PLGA and MTX-loaded PLGA NPs samples were assayed by FTIR (Spectrum 400, PerkinElmer, Waltham, MA, USA). FTIR spectra showed chemical group of each component and interaction between components. FTIR assays were conducted with scanning at 380–4000 cm^−1^.

XRD assays of MTX, PLGA, physical mixture of MTX/PLGA, and MTX-loaded PLGA NPs were performed using an X-ray diffractometer (Empyrean, Malvern Panalytical, Almelo, The Netherlands). The scanning range of 2θ was from 5° to 90°.

DSC studies of MTX, PLGA, physical mixture of MTX/PLGA, and MTX-loaded PLGA NPs were conducted using a differential scanning calorimeter (DSC823e, Mettler-Toledo, Greifensee, Switzerland). DSC thermograms provided information for physical characteristics of samples including crystalline or amorphous form. Samples were scanned from 0°C to 300 °C at a rate of 10 °C/min.

#### 3.3.4. Morphological Studies

The morphology of MTX-loaded NPs was studied with a field-emission scanning electron microscope (FE-SEM, JSM-7500F, JEOL Ltd., Tokyo, Japan). Prepared NPs samples were coated with platinum (~20 nm thick) using an Ion Sputter (JFC-1100, JEOL Ltd., Tokyo, Japan) for 5 min at 20 mA. These NPs were observed at an accelerating voltage of 15 kV.

#### 3.3.5. In Vitro Release Studies

For in vitro release studies of MTX, prepared NPs and free MTX as control formulation were investigated at different pH (1.2, 6.8, and 7.4) with dialysis method [70,71]. Briefly, 10 mg of prepared NPs was dissolved in 10 mL of each medium. Then 1 mL of the solution was put in a dialysis tube (molecular weight cut-off 12 kDa, Sigma-Aldrich, St. Louis, MO, USA) which was placed into a 50 mL screw-capped tube containing 10 mL of each dissolution medium. These tubes were constantly shaken at a rate of 50 rpm in a shaking water bath at 37 °C. Whole-media were changed to prevent drug saturation during the study. At predetermination time intervals of 1, 2, 4, 6, 8, 12, 24, 48, 72, 96, and 120 h after incubation, whole media (10 mL) were taken and replaced with equal volume of fresh medium (10 mL). The amount of released MTX was determined by UPLC-MS/MS.

#### 3.3.6. In Vitro Cytotoxicity Studies

MCF-7 cells (human breast cancer cell line) and CWR22Rv1 cells (human prostate cancer cell line) were cultured in RPMI 1640 medium supplemented with 10% FBS and 1% penicillin/streptomycin in a humidified atmosphere of 5% CO_2_ at 37 °C.

##### Trypan Blue Assay

Cytotoxic effects were determined by trypan blue dye exclusion method. Cells were seeded into 9 cm Petri dishes and allowed to adhere for 24 h. Cells were incubated with free MTX, blank NPs, MTX-loaded NPs, or PBS alone as control. After 96 h, each plate was treated with 0.4% trypan blue in PBS. The morphology of each cell was observed using an optical microscope.

##### 3-(4,5-dimethylthiazol-2-yl)-5-(3-carboxymethoxyphenyl)-2-(4-sulfophenyl)-2H-tetrazolium (MTS) Assay

Cell proliferation was quantified with MTS assay using CellTiter 96^®^ AQueous One Solution (Promega, Madison, WI, USA). Cells were seeded in 96-well plates and incubated for attachment. After incubation for 24 h, cells were treated with free MTX, blank NPs, or MTX-loaded NPs. Cells incubated with PBS alone were used as controls. Cell viability was observed before treatment and at 24, 48, 96, 108, and 144 h after treatment of each sample. The assay was carried out according to the manufacturer’s protocol. Briefly, 20 μL of MTS reagent was added to each well at the end of the incubation time and cells were incubated at 37 °C for 2 h. Absorbance was measured at 490 nm using a microplate reader (PowerWave HT, BioTek, Winooski, VT, USA). Background absorbance at 650 nm was subtracted. Cell viability was calculated with the following equation:

(3) $$\mathrm{Cell}\;\mathrm{viability}\;(\%)\;=\;\frac{{\mathrm{Absorbance}}_{\left(490-650\right)}\;\mathrm{of}\;\mathrm{sample}\;\mathrm{cells}}{{\mathrm{Absorbance}}_{\left(490-650\right)}\;\mathrm{of}\;\mathrm{control}\;\mathrm{cells}}\times 100$$

##### Cellular Folates Analysis

As mentioned above, MTX can inhibit intracellular DHFR which catalyzes the conversion of DHF to THF, subsequently leading to cancer cell death. To assess the cytotoxic effect of MTX-loaded PLGA NPs, we quantified cellular concentration of DHF and THF after treating cells with free MTX, blank NPs, or MTX-loaded NPs. Control cells were treated with PBS alone. The assay was conducted with modification of previously reported protocols [72,73] using UPLC-MS/MS system. Briefly, cells were plated onto 9 cm plates and grown for 24 h. These cells were cultured with free MTX, blank NPs, or MTX-loaded NPs (22 μM of MTX). After incubation for 144 h, cells on each plate were washed three times at room temperature with PBS (pH 7.4) before extraction. MTX, DHF, and THF were extracted from cultured cells by aspirating PBS washing solution and the media, and immediately adding 1 mL of ice-cold water−methanol (5:5, *v/v*) containing 25 mM sodium ascorbate, 0.1% (*v/v*) 2-mercaptoethanol and 25 mM ammonium acetate at pH 7.0. Plates were kept on ice (2 °C) for 30 min. Cells were scraped and the resulting mixture of cells and solvent were transferred to centrifuge tubes. A 10 μL of IS solution (10 μg/mL of phenacetin dissolved in methanol) was also added to each tube. Tubes were heated to 70°C for 5 min to fully denature proteins, and precipitates were removed by centrifugation at 20,000× *g* for 5 min at 5 °C. Supernatants were dried under nitrogen gas flow and resuspended in 450 μL of potassium phosphate buffer (50 mM with 30 mM ascorbic acid and 0.5% 2-mercaptoethanol at pH 7.0). Rat serum containing folate conjugase was treated with activated charcoal to remove endogenous folate. Then 25 μL of rat serum pretreated with activated charcoal was added to the samples in order to deconjugate polyglutamates of intracellular folates followed by incubation at 37 °C for 2 h. After that, samples were placed in a heated water bath (70 °C) for 5 min and then centrifuged at 20,000 × g for 15 min at 5 °C. To clean up samples, supernatants were transferred to a 10 kDa molecular weight cut-off membrane filter (Microcon^®^ Centrifugal Filters, Merck Millipore, Burlington, MA, USA) and centrifuged at 20,000× *g* for 30 min at 20 °C. The solution at the bottom of the tube was then injected into a UPLC-MS/MS system.

#### 3.3.7. In Vivo Studies

Animal experiments were approved by Chonnam National University Animal Experimental Ethics Committee, Republic of Korea (CNU IACUC-YB-2017–47). Sprague−Dawley male rats (8 weeks, 240–260 g) were purchased from Damul Science (Daejeon, Republic of Korea). All rats were housed separately in metabolic cages in an environmentally controlled room (23°C ± 1°C, 12 h light/dark cycle) with free access to food and water before experiments. Each rat was fasted overnight before drug administration. These rats were divided into four groups (five rats in each group): (1) free MTX for oral administration, (2) MTX-loaded NPs for oral administration, (3) free MTX for IV administration, and (4) MTX-loaded NPs for IV administration. For IV administration, groups 3 and 4 were cannulated at femoral vein and artery with polyethylene tube (PE-50, Becton Dickinson, Sparks, MD, USA) under light ether anesthesia. Prior to experiment, anesthetized rats were kept in restraining cages under normal housing conditions for 1 h until recovery from anesthesia. Single dose (5 mg/kg as MTX) of each formulation was given to rats orally or intravenously. The dose of MTX used in clinical trials varied according to various factors, including the type of disease, age, concomitant medication, and so on [74]. The dose of MTX used in this animal experiment was set considering both clinical doses normally used and solubility of the drug in the solvent. Blood samples were collected via jugular vein or femoral artery before administration (0 h) and at 0.25, 0.5, 1, 2, 4, 6, 8, 12, 24, and 36 h after administration. They were placed into microtubes (Axygen, Union City, CA, USA) containing 5 μL of heparin solution (25,000 IU/5 mL, Choongwae Pharm. Co., Seoul, Republic of Korea) to prevent blood clotting. Plasma was immediately separated by centrifuging blood samples at 8000 rpm for 10 min and stored at −70 °C until analysis.

To evaluate lymphatic and tissue delivery of MTX, rats were divided into four groups (five rats in each group) and administered with each formulation as mentioned above. At 2.5 h after administration, whole blood was taken via the abdominal aorta. Targeted lymphatic system (mesenteric and axillary lymph nodes), immune tissues (spleen and thymus), and non-targeted normal tissues (kidney and liver) were then isolated and weighed. Liver samples were put in an equal ratio (*w/v*) of PBS (pH 7.4). Remaining biological samples were placed in 1 mL of PBS. Biological samples were homogenized for 1 min. Suspensions were then stored at −70 °C until analysis.

#### 3.3.8. Determination of MTX in Rat Plasma, Lymph Node and Tissues

Quantitative analysis of MTX in rat biological matrices was conducted using a Shimadzu Nexera X2 Series UPLC system (Shimadzu Corp., Kyoto, Japan) coupled with a Shimadzu 8040 mass spectrometer. Optimized chromatographic separation of MTX was performed with a Phenomenex KINETEX core-shell C18 column at a column temperature of 40 °C. The mobile phase consisted of 0.1% (*v/v*) formic acid aqueous solution (mobile phase A) and 100% acetonitrile (mobile phase B) with gradient elution at flow rate of 0.3 mL/min. Elution program consisted of 0–0.5 min (10% B), 0.5–0.8 min (10–90% B), 0.8–3.0 min (90% B), 3.0–3.01 min (90–10% B), and 3.01–4.0 min (10% B). Total run time was 4 min per sample. All analytical procedures were evaluated with positive electrospray ionization. Quantification was achieved using MRM modes at m/z 454.7→308.1 for MTX and at m/z 180.0→110.10 for IS.

To prepare standard stock solutions at 1 mg/mL, MTX and IS were weighed accurately and dissolved in methanol/water (8/2, *v/v*) and 100% methanol, respectively, prior to make each working solution and stored at −20 °C. Standard working solutions of MTX (1, 10, 50, 100, 500, 1000, 5000, and 10,000 ng/mL) and IS (100 ng/mL) were diluted with methanol/water (8/2, *v/v*) and 100% methanol, respectively, from its standard stock solution. Calibration standards were prepared by adding and mixing each diluted working solution into blank rat biological samples to obtain final concentrations of MTX ranging from 0.1 to 1000 ng/mL. To examine accuracy and precision of UPLC-MS/MS method, quality control samples at four levels (0.1, 20, 400 and 800 ng/mL) were similarly prepared.

Samples were extracted by liquid-liquid extraction method using ethyl acetate. Protein was precipitated by protein precipitation method using acetonitrile. Then 100 μL of rat biological sample was added to 10 μL of IS solution (phenacetin of 100 ng/mL). The mixed sample was added to 1000 μL acetonitrile-ethyl acetate (9/1, *v/v*), vortexed for 5 min and centrifuged at 13,500× *g* for 5 min. Then 1000 μL of the supernatant organic layer was dried gently with a nitrogen centrifugal evaporator at 40 °C. The dried residue was reconstituted with 50 μL of mobile phase and vortexed for 5 min. After centrifugation for 5 min at 13,500× *g*, 5 μL of aliquot was injected for UPLC-MS/MS analysis.

#### 3.3.9. Pharmacokinetic Analysis and Targeting Delivery Evaluation

PK parameters of MTX formulations after oral or intravenous administration to rats were estimated by noncompartmental analysis using WinNonlin^®^ software (version 8.1, Pharsight^®^, a Certara™ Company, CA, USA). C_max_ and T_max_ were individually calculated using plasma concentration-time curve. AUC from 0 to t h after administration (AUC_0-t_) was calculated by the linear trapezoidal rule. AUC from 0 to infinity (AUC_0-∞_) was calculated as AUC_0-t_ + C_t_/k, where C_t_ was the last measurable concentration and k was the elimination rate constant at terminal phase. The half-life was calculated to be 0.693/k and systemic clearance was determined as dose/AUC_0-∞, IV_. V_d_ was calculated as dose/k·AUC_0-∞, IV_. Absolute oral bioavailability for each formulation was calculated as AUC_0-∞, oral_/AUC_0-∞, IV_ × 100 and relative bioavailability of PLGA NPs was calculated as AUC_0-∞, PLGA NP_/AUC_0-∞, free-MTX_ × 100. F_ab_ was the amount of the drug available to the body or system in the same formulation and F_rb_ was used to compare bioavailability between the formulation and the standard. Targeting efficiency of MTX to the lymphatic system was calculated as the ratio of MTX concentration in axillary and mesenteric lymph node to MTX concentration in rat plasma at 2.5 h after administration of each formulation. All data are expressed as mean ± standard deviation.

#### 3.3.10. Statistical Analysis

We analyzed data for statistical significance using Student’s *t*-test. Statistical significance was set at *p* < 0.05. The Statistical Package for the Social Sciences (SPSS) software version 23 (IBM, Armonk, NY, USA) was used for all statistical analyses.

## 4. Conclusions

MTX-loaded PLGA NPs could be prepared easily by double emulsion solvent evaporation method. At optimal conditions (0.5% (*w/v*) of PLGA, 1% (*w/v*) of PVA, 1:3 (*v/v*) of oil−water phase volume ratio), mean particle size, zeta potential, and drug EE of MTX-loaded NPs were 163.7 ± 10.25 nm, −18.5 ± 2.28 mV, and 93.34 ± 0.51%, respectively. In vitro cytotoxicity studies resulted that prepared NPs had higher antiproliferative effects against cancer cells compared to the control group. These NPs exhibited sustained in vitro release profiles relative to free MTX at various pH conditions. In vivo studies showed that these prepared PLGA NPs had distinctly different PK characteristics from free MTX, with high lymphatic efficiency but low migration and accumulation in kidney and liver. Therefore, we could conclude that these prepared MTX-loaded NPs with both administration routes can be used to improve lymphatic targeting delivery of MTX. Also, it seems possible to reduce their delivery to normal tissues such as kidney and liver known to be related to major adverse effects of MTX.

## Figures and Tables

**Table 1:** Effect of poly(d,l-lactide-co-glycolide) (PLGA) concentration on mean particle size (*n* = 5, mean ± SD).

PLGA Conc. (%, *w/v*)	PVA Conc. (%, *w/v*)	Oil:Water Phase Ratio (*v/v*)	Nanoparticle Size (nm)
0.5	10	1:3	110.5 ± 8.38
1	10	1:3	117.3 ± 9.54
2	10	1:3	152.7 ± 12.17 *
4	10	1:3	276.7 ± 19.25 *
6	10	1:3	456.2 ± 23.03 *
8	10	1:3	873.8 ± 30.11 *

* *p* < 0.05 compared to the group with 0.5% *w/v* of PLGA.

**Table 2:** Effect of poly vinyl alcohol (PVA) concentration on mean particle size (*n* = 5, mean ± SD).

PVA Conc. (%, *w/v*)	PLGA Conc. (%, *w/v*)	Oil–Water Phase Ratio (*v/v*)	Nanoparticle Size (nm)
1	4	1:3	135.5 ± 7.92
2.5	4	1:3	167.9 ± 10.33 *
5	4	1:3	212.8 ± 13.20 *
7.5	4	1:3	269.5 ± 15.09 *
10	4	1:3	313.8 ± 15.86 *
15	4	1:3	404.3 ± 20.42 *

* *p* < 0.05 compared to the group with 1% *w/v* of PVA.

**Table 3:** Effect of oil–water phase volume ratio on mean particle size (*n* = 5, mean ± SD).

Oil:Water Phase Ratio (*v/v*)	PLGA Conc. (%, *w/v*)	PVA Conc. (%, *w/v*)	Nanoparticle Size (nm)
1:1	0.5	1	332.3 ± 15.56
1:2	0.5	1	201.2 ± 20.25 *
1:3	0.5	1	103.0 ± 11.74 *
1:4	0.5	1	199.3 ± 14.38 *
1:5	0.5	1	254.3 ± 18.31 *
1:6	0.5	1	315.5 ± 17.90 *

* *p* < 0.05 compared to the group with 1:1 volume ratio of oil and water phase.

**Table 4:** Physicochemical and encapsulation characteristics of nanoparticles (NPs) (*n* = 5, mean ± SD).

Methotrexate Amount (mg)	PLGA Conc. (%, *w/v*)	PVA Conc. (%, *w/v*)	Oil–Water Phase Ratio (*v/v*)	Nanoparticle Size (nm)	Zeta Potential (mV)	Encapsulation Efficacy (%)	Drug Loading (%)
0	0.5	1	1:3	103.0 ± 11.74 *	−20.4 ± 1.54	-	-
3	0.5	1	1:3	163.7 ± 10.25	−18.5 ± 2.28	93.34 ± 0.51	15.45 ± 0.34
6	0.5	1	1:3	206.1 ± 18.14 *	−17.3 ± 2.63	90.26 ± 0.63 *	13.10 ± 0.48 *
9	0.5	1	1:3	258.3 ± 15.32 *	−19.7 ± 3.10	91.12 ± 0.49 *	14.33 ± 0.31 *
15	0.5	1	1:3	299.2 ± 17.85 *	−18.9 ± 2.98	88.28 ± 1.02 *	11.07 ± 0.85 *
20	0.5	1	1:3	351.4 ± 20.10 *	−17.8 ± 3.24	84.31 ± 1.90 *	9.69 ± 1.36 *

* *p* < 0.05 compared to the 3 mg of MTX group.

**Table 5:** Concentrations of dihydrofolate (DHF), tetrahydrofolate (THF), and MTX in CWR22Rv1 and MCF-7 cells after incubating cells with free MTX, blank NPs, or MTX-loaded NPs for 144 h (*n* = 3, mean ± SD).

	Intracellular Concentration (μmol/L)			
	Free MTX	Blank NPs	MTX-Loaded NPs	Control
**CWR22Rv1**				
DHF	4.85 ± 1.30 *	1.05 ± 0.33	8.42 ± 2.17 *^,^^#^	1.14 ± 0.38
THF	0.89 ± 0.21 *	2.10 ± 0.64	0.44 ± 0.15^*^	1.92 ± 0.49
MTX	1.04 ± 0.27	-	2.52 ± 0.61^#^	-
**MCF-7**				
DHF	2.74 ± 1.12 *	1.50 ± 0.54	4.03 ± 1.76 *^,^^#^	1.46 ± 0.60
THF	1.47 ± 0.49 *	2.62 ± 0.79	1.02 ± 0.34 *	2.59 ± 0.84
MTX	0.95 ± 0.31	-	2.38 ± 0.55 ^#^	-

* *p* < 0.05 compared to only medium treated as control. ^#^ *p* < 0.05 between prepared PLGA NPs and free MTX groups.

**Table 6:** Pharmacokinetic parameters of MTX after administration of MTX-loaded NPs or free MTX in rats (*n* = 5, mean ± SD).

Parameters	Free MTX (5 mg/kg)		PLGA NPs (5 mg/kg as MTX)	
	Oral	Intravenous	Oral	Intravenous
AUC_0-t_ (ng·h/mL)	59.73 ± 23.74	375.46 ± 114.61	152.59 ± 7.32 *	728.17 ± 84.37 *
AUC_0-∞_ (ng·h/mL)	60.33 ± 23.19	376.09 ± 115.31	153.29 ± 7.34 *	730.33 ± 85.06 *
t_1/2_ (h)	1.53 ± 0.54	1.56 ± 0.56	3.01 ± 0.28 *	4.94 ± 0.15 *
C_max_ (ng/mL)	16.95 ± 2.24	-	31.19 ± 5.15 *	-
T_max_ (h)	0.75 ± 0.25	-	0.92 ± 0.14	-
CL (L/h·kg)	-	3.53 ± 1.03	-	1.73 ± 0.21 *
V_d_ (L/kg)	-	29.84 ± 4.59	-	55.96 ± 9.76 *
F_ab_ (%)	16.04	-	20.99 *	-
F_rb_ (%)	-	-	254.09	194.19

* *p* < 0.05 between prepared PLGA NPs and free MTX groups.
